# A description of nesting behaviors, including factors impacting nest site selection, in black‐and‐white ruffed lemurs (*Varecia variegata*)

**DOI:** 10.1002/ece3.4735

**Published:** 2019-01-01

**Authors:** Andrea L. Baden

**Affiliations:** ^1^ Department of Anthropology Hunter College of the City University of New York New York New York; ^2^ Departments of Anthropology and Biology The Graduate Center of the City University of New York New York New York; ^3^ The New York Consortium in Evolutionary Primatology New York New York

**Keywords:** communal breeding, crèche, infant parking, lemuriform, Madagascar, strepsirrhines

## Abstract

Nest site selection is at once fundamental to reproduction and a poorly understood component of many organisms’ reproductive investment. This study investigates the nesting behaviors of black‐and‐white ruffed lemurs, *Varecia variegata*, a litter‐bearing primate from the southeastern rainforests of Madagascar. Using a combination of behavioral, geospatial, and demographic data, I test the hypotheses that environmental and social cues influence nest site selection and that these decisions ultimately impact maternal reproductive success. Gestating females built multiple large nests throughout their territories. Of these, females used only a fraction of the originally constructed nests, as well as several parking locations as infants aged. Nest construction was best predicted by environmental cues, including the size of the nesting tree and density of feeding trees within a 75 m radius of the nest, whereas nest use depended largely on the size and average distance to feeding trees within that same area. Microhabitat characteristics were unrelated to whether females built or used nests. Although unrelated to nest site selection, social cues, specifically the average distance to conspecifics’ nest and park sites, were related to maternal reproductive success; mothers whose litters were parked in closer proximity to others’ nests experienced higher infant survival than those whose nests were more isolated. This is likely because nesting proximity facilitated communal crèche use by neighboring females. Together, these results suggest a complex pattern of nesting behaviors that involves females strategically building nests in areas with high potential resource abundance, using nests in areas according to their realized productivity, and communally rearing infants within a network of nests distributed throughout the larger communal territory.

## 1 INTRODUCTION

Nests are widely recognized for their importance to reproduction (e.g., Wilson, 1998; Madsen & Shine, 1999; Rauter, Reyer, & Bollmann, 2002; Benson, Lotz, & Jansen, 2008; Cudworth & Koprowski, 2011; Mainwaring, Deeming, Jones, & Hartley, 2014; Mainwaring, Hartley, Lambrechts, & Deeming, 2014), and yet nesting *behaviors* (i.e., nest site selection, construction, use, and reuse) remain an understudied component of many organisms’ reproductive investment. Among vertebrates, nesting is taxonomically widespread (most birds, many amphibians, fish, mammals, and reptiles), takes on many forms, and serves multiple purposes (e.g., reproduction, food storage, predator avoidance, sexual signaling; reviewed in Hansell, 2000, 2005). Nests can range in complexity, from simple structures like the detritus mounds of megapodes (Jones, Dekker, & Roselaar, 1995), the elliptical mud chambers of South American hylids (e.g., *Aplastodiscus perviridis*: Haddad, Faivovich, & Garcia, 2005), and the stick platforms of many birds (e.g., doves, pigeons: Goodwin, 1983; owls: Wu et al., 2015; raptors: Canal, Mulero‐Pázmány, Negro, & Sergio, 2016), to complex burrow systems (e.g., deer mice, Lewarch & Hoekstra, 2018; mole rats: Lövy et al., 2015), intricately woven nests (e.g., black‐headed weaver birds: Collias&Collias, 1959, 1984), and nests with elaborate and/or colorful displays (e.g., bower birds: Borgia, 1985). Still other animals do not construct their own nests at all, instead using the abandoned nests of heterospecifics to bear and rear their young (e.g., “secondary modifiers” or “simple occupants” sensu Kinlaw (1999); e.g., burrowing owls: Butts & Lewis, 1982; golden jackals: Mukherjee, Kumara, & Bhupathy, 2018).

Despite their diversity in form and function, most nests play a fundamental role in reproduction, which is to provide optimal conditions in which to lay eggs and/or raise dependent offspring (Heenan, 2013; Mainwaring et al., 2017; Mainwaring, Deeming, et al., 2014; Mainwaring, Hartley, et al., 2014). In the last several decades, where and how organisms nest has received considerable attention (reviewed in Hansell, 2005; Refsnider & Janzen, 2010; Mainwaring, Deeming, et al., 2014; Mainwaring, Hartley, et al., 2014; Mainwaring et al., 2017). However, investigations have focused primarily on avian taxa, particularly the cavity nesting birds—especially small passerines—whose reliance on tree holes has allowed researchers to monitor and experimentally manipulate eggs, nestlings, and nesting environments in the wild via nest boxes (Lambrechts et al., 2010). Comparatively fewer studies have investigated nesting behavior in other classes of animals (Barber, 2013); nevertheless, the current literature reveals that vertebrate nesting strategies are diverse and often convergent, with nesters relying on cues from their physical and social environments to make decisions about the placement, construction, use, and reuse of nests.

Nest site selection is a critical first step in the nesting process and is essential for ensuring optimal microhabitat conditions for incubation and infant rearing (Durant, Hopkins, Hepp, & Walters, 2013; Hansell, 2005). Although the specifics of microhabitat preference vary, their functions can be generalized to one of only a handful of roles. For instance, many animals exhibit preference for sites that are able to provide sound structural support to both mother and offspring: golden mice preferentially nest in areas of high stem density to provide increased attachment points for nests (Wagner, Feldhamer, & Newman, 2000); gray squirrels nest in large trees with thick basal areas to make nests less prone to wind damage (Gregory, Vander Haegen, Chang, & West, 2010); and wolverines seek habitats with suitable denning structures (e.g., boulders, snow drifts) to keep dens warm and dry (May et al., 2012). Likewise, animals prefer sites that offer protection from the elements, while also allowing them to avoid and evade predators (and parasites). Tortoises (Pignati, Fernandes, Miorando, Ferreira, & Pezzuit, 2013), porcupines (Mukherjee, Kumara, & Bhupathy, 2017), and European shags (Barros, Romero, Munilla, Perez, & Velando, 2016) select relatively high elevation sites characterized by good drainage to avoid incidents of nest and/or burrow flooding, while American marten (Ruggiero, Pearson, & Henry, 1998), gray squirrels (Cudworth & Koprowski, 2011), and jackals (Mukherjee et al., 2018) select nest sites with plentiful escape routes and/or nearby refuges. It is also common for animals to nest in well‐insulated areas, such as those with thick vegetation cover (golden mice: Wagner et al., 2000); optimal sun exposure (badgers: Davis, 2005; Márton et al., 2016); and/or deep, well‐lined cavities (birds: Mazgajski, 2003; Hilton, Hansell, Ruxton, Reid, & Monaghan, 2004; Mainwaring, Deeming, et al., 2014; Mainwaring, Hartley, et al., 2014). Some studies have found that “high quality” sites are often also located in proximity to valuable resources, such as preferred food items (gray squirrels: Cudworth & Koprowski, 2011), or prey (red fox: Carter, Luck, & Wilson, 2012; Indian fox: Punjabi, Chellam, & Vanak, 2013).

Beyond habitat characteristics, nest prospectors may also use direct and/or indirect social cues, such as the presence (Hartman, Ackerman, Takekawa, & Herzog, 2016; Kivelä et al., 2014; Podofillini et al., 2018), quality (e.g., male rank: Ramsay, Otter, & Ratcliffe, 1999), and/or prior clutch success of conspecifics (Kivelä et al., 2014), and the presence, density, and/or behavior of heterospecifics (i.e., “heterospecific attraction”; e.g., Mönkkönen et al., 1990; Seppänen & Forsman, 2007; Loukola, Seppänen, & Forsman, 2012; Avarguès‐Weber, Dawson, & Chittka, 2013; Seppänen, Forsman, Mönkkönen, Krams, & Salmi, 2011; but see Slagsvold & Wiebe, 2017) as indicators of habitat quality, also known as “public information” (reviewed in Danchin, Giraldeau, Valone, & Wagner, 2004). In cooperative breeders, nest site selection may also depend on the availability of (and proximity to) helpers or other breeding females (Hatchwell, Russell, Fowlie, & Ross, 1999; Lawton & Lawton, 1980).

Of course, none of these cues are mutually exclusive and nesting behaviors are likely motivated by several factors simultaneously. When selecting a nest site, animals must therefore find the best compromise between their preferred microhabitats, the risk of predation, and the availability of resources nearby the nest (Cudworth & Koprowski, 2011; Juškaitis, Balčiauskas, & Šiožinyte, 2013). Accordingly, nest site selection is often a series of trade‐offs. For instance, Australian turtles (*Emydura macquarii*) forego their preferred open microhabitat to minimize nest predation risk by locating nests away from shore (Spencer & Thompson, 2003), while song thrushes (*Turdus philomelos*) nest in trees with intermediate foliage cover, accepting an increase in predation for better visibility (Götmark, Blomqvist, Johansson, & Bergkvist, 1995).

Once nest sites are selected, construction can begin. As noted earlier, nests range in diversity from simple to complex, and vary in their placement (e.g., arboreal, terrestrial, fossorial), structure (e.g., burrows, platforms, cups), and materials used (e.g., clay, branches, leaves) (Hansell, 2005). The process of nest building can take hours, days, weeks, and even months, with the degree of differential parental investment being equally as diverse (i.e., exclusive maternal, paternal, or biparental investment) (Hansell, 2005; Soler, Møller, & Soler, 1998). In most vertebrates, one or both parents invest in a single nest per breeding attempt, such that unless the nest is disturbed (e.g., Beckmann, Biro, & Martin, 2015; Flegeltaub, Biro, & Beckmann, 2017), whether it is used goes more or less without question. But in some, rare cases, breeders build multiple nests from which to choose for reproduction (e.g., marsh wren: Verner & Engelsen, 1970; European wren: Garson, 1980; Australian reed warblers: Berg, Beintema, Welbergen, & Komdeur, 2006; raptors: Ontiveros, Caro, & Pleguezuelos, 2008; Pallas's cats: Ross, Kamnitzer, Munkhtsog, & Harris, 2010). Whether nests are used once and abandoned, used repeatedly within and across breeding seasons (i.e., high nest site fidelity), and/or are used singly or by multiple nesting individuals is equally variable (e.g., Ross et al., 2010; Lovich et al., 2014; Robert et al., 2014).

Nest construction and maintenance can be both temporally and energetically costly to parents (e.g., Collias & Collias, 1984; Berg et al., 2006; Tomás et al., 2006; Mainwaring & Hartley, 2013; Smith, Harrison, Martin, & Reynolds, 2013), and decisions during nest use can have significant reproductive consequences in terms of infant growth and survival (Chalfoun & Schmidt, 2012; Martin, 1998; Resetarits, 1996; Zhao, Hu, Liu, Chen, & Sun, 2016). For example, reduced nest attendance in rats has long‐term effects on infant malnutrition (Massaro, Levitsky, & Barnes, 1974), whereas communal nest use in several taxa results in higher infant survival until weaning (e.g., König, 1997; Baden, Wright, Louis, & Bradley, 2013; but see Hayes, 2000). Thus, the location and design of nests, as well as the subsequent nesting behaviors, are all decisions critical to nestling survival and long‐term parental reproductive success (reviewed in Martin, 1998; Refsnider & Janzen, 2010; Mainwaring & Hartley, 2013). Given the complexities and costs associated with the myriad nesting behaviors described above, it stands to reason that nesting behaviors should be under strong selective pressure and should be included among the life‐history traits of critical importance for many species (Chalfoun & Schmidt, 2012; Hartman et al., 2016; Martin, 1998; Resetarits, 1996). Moreover, although studied in great detail in avian taxa, investigations of the patterns, processes, and adaptive consequences of nesting behaviors are lacking in other vertebrate taxa.

Here, I describe the nesting behaviors—including nest site selection, construction, use, and reuse—of black‐and‐white ruffed lemurs, *Varecia variegata*, a litter‐bearing primate with a communal breeding reproductive system (Baden et al., 2013; Morland, 1990; Vasey, 2007). Ruffed lemurs (Genus *Varecia*) are relatively large bodied (3.5–4.6 kg: Baden, Brenneman, & Louis, 2008), diurnal strepsirrhines restricted to the low‐ to mid‐altitude rainforests of eastern Madagascar (Morland, 1991; Balko, 1998; Ratsimbazafy, 2002; Vasey, 2003; Baden, 2011). Ruffed lemurs are highly frugivorous (Balko & Underwood, 2005; Erhart, Tecot, Grassi, 2018; Wright et al., 2011) and form large, stable social “communities” to cooperatively defend preferred fruit resources (reviewed in Baden, Webster, & Kamilar, 2016). Group movement, however, is not coordinated, and members of a social community exhibit extensive fission–fusion social dynamics (Baden et al., 2016).

As with most Malagasy strepsirrhines, ruffed lemurs are strict seasonal breeders (Bogart Cooper, & Benirschke, 1977; Bogart, Kumamoto, & Lasley, 1977; Boskoff, 1977; Foerg, 1982; Morland, 1993; Rasmussen, 1985) and are the only diurnal primates known to bear litters of 2–3 offspring during these seasonal reproductive events (Baden et al., 2013; Foerg, 1982; Rasmussen, 1985). Offspring are born altricial (e.g., eyes closed, incapable of clinging) and mothers must carry infants orally until they are able to move about on their own (~10 weeks; Baden et al., 2013). Because of the constraints imposed by litters of relatively underdeveloped young, mothers park infants in nests and tree tangles until capable of independent travel (Baden, 2011; Baden et al., 2013; Morland, 1990; Vasey, 2007).

While *Varecia* nest use has been previously documented (Baden et al., 2013; Klopfer & Dugard, 1976; Morland, 1990; Pereira, Klepper, & Simons, 1987; Vasey, 2007), details of their nesting behaviors (i.e., nest site selection, construction, use, and reuse) have yet to be fully described. Moreover, the potential benefits of nest site selection to infant survival and maternal reproductive success have yet to be addressed.

Here, I describe the nesting behaviors of seven parous black‐and‐white ruffed lemur females during the only reproductive season observed in 6 years of continuous observation. The overarching goal of this study was to examine potential relationships between environmental and social cues, nesting behaviors, and infant survival. Specifically, I ask four main questions: (a) Do nest sites differ from control sites? (b) Of the nests constructed during gestation, what predicts whether nests are used? (c) Of the nests that are used, what predicts the occurrence and frequency of reuse and/or crèching? And finally, (d) can nest site characteristics and/or nesting behaviors explain maternal reproductive success? To address these questions, I describe nest construction, including the frequency and duration of nest building behaviors, and the total number of nests constructed, and characterize their nesting environment, including the locations of nesting sites and microhabitat characteristics relative to their larger overall home range.

Based on what is known of nest site selection in other organisms, I expected ruffed lemur nest sites to differ from control sites in ways that might provide sound structural support to nests (e.g., relatively larger basal area and/or crown diameter), protection from the elements and/or predators (e.g., denser canopy cover to protect against aerial predators and/or rain exposure; reduced ground cover to improve visibility necessary for terrestrial predator avoidance; and/or reduced water cover to avoid drowning if/when infants fall from the nest), and/or access to high‐quality food resources for mothers during periods of nest use (e.g., number and/or density of feeding trees in proximity to the nest). Similarly, I expected females to preferentially use, reuse, and/or share nests for these same qualities, preferentially using the safest, most structurally sound nests more often than others. In addition, because of their communal breeding system, I expected patterns of nesting and parking behaviors to be motivated by social factors. Thus, in addition to environmental characteristics, I also expected females to preferentially use sites located in closer proximity to other females. Finally, operating under the assumption that there is strong selection for nesting behaviors that will improve individual reproductive success, I expected to find a relationship between maternal nesting patterns and infant survival to locomotor independence.

## 2 METHODS

### 2.1 Ethical note

Research protocols were in compliance with and permission was granted by Stony Brook University IACUC #2005‐20081449 and Madagascar’s National Parks (ANGAP/MNP).

### 2.2 Study site and species

Data were collected from one wild, habituated black‐and‐white ruffed lemur (*V. variegata*) community in Mangevo (21°22′60″S, 47°28′0″E), a mid‐elevation primary rainforest site located in Parcel III of Ranomafana National Park (RNP), Madagascar (Wright, 1992; Wright et al., 2012). Data were collected over a 6‐year period (2005–2010). Changes in group demography (births, deaths, emigrations, immigrations) were monitored during monthly surveys between 2005 and 2010, while detailed behavioral observations were collected continuously during a 17‐month period (August 2007–December 2008). Reproduction was only observed during 2008. Thus, a majority of the data presented herein are limited to a six‐month period that spanned all of gestation and nesting/parking (July–December 2008, *n* = 3,450 hr). This study concluded when infant nesting/parking ceased (i.e., the onset of infant independent travel).

Prior to the onset of behavioral sampling, all members of the Mangevo ruffed lemur community were given subcutaneous AVID^®^ microchips and individually identified via radio‐collars and/or unique collar‐tag combinations. Animal captures were performed under veterinary supervision following established protocols (Glander, 1993). At the time of the study, the community included 24 adults and subadults (eight adult females, 11 adult males, five subadult males). Nineteen infants were born in the 2008 birth season and were present from October to December 2008, when the study ended. Of the study subjects, five females and three males were radio‐collared and targeted for regular follows. Individuals with collar‐tags (but no radio‐collars, *n* = 16) were opportunistically targeted for focal follows. Two of seven females included in this study used the periphery of the communal territory during most of the year and were not the subjects of focal observations; however, in the 10 weeks following parturition, both females and their litters were contacted regularly and were often found associating and nesting their litters communally with focal individuals. We were unable to quantify nesting behaviors for these females; however, regular observations of co‐nesting associations between these two females and other parous females within the study allowed us to characterize focal females’ nests as single or shared.

### 2.3 Data collection

#### 2.3.1 Observational data

Two teams of four observers each selected a subject at random for all‐day behavioral observations (i.e., two animals were followed daily), during which time each observer per team was responsible for one of the following tasks: instantaneous focal animal sampling, continuous focal animal sampling, instantaneous focal nest sampling, and/or locating and tracking animals (Altmann, 1974). Focal subjects were located at the beginning of each observation period via radio‐telemetry. Only independent individuals (adults and subadults) were targeted for follows. Observations were rotated among individuals daily, and sampling was distributed evenly among subjects. Observations ranged from 8 to 11 hr, depending on seasonal differences in day length and the time it took observers to locate animals at dawn. Teams were routinely checked for interobserver agreement to ensure comparability of data (Martin & Bateson, 2007).

One observer used instantaneous sampling to record the focal subject's behavioral state at 5‐min intervals (feeding, foraging, resting, traveling, social, other), as well as its subgroup size (i.e., individuals within 50 m of one another who exhibited behavioral coordination), composition, and cohesion (see Baden et al., 2016 for details). If the focal animal was observed feeding during a scan, we recorded the Tree ID (if tagged; see below), species, part eaten, and phenological stage (e.g., ripe vs. unripe fruit, young vs. mature leaves).

A second observer simultaneously used continuous recording to contextualize the focal subject's behaviors, documenting all vocalizations, affiliative, aggressive, and socio‐sexual interactions (including anogenital inspections, mounts, and mating events), and details of nesting behaviors, including the identities of nest builder(s), and the duration and details of nest construction and nest use (e.g., nesting materials used, method of nest construction, frequency of nest transfers).

During the 10 weeks following parturition (mid‐October to December 2008), observational protocols were supplemented with all‐day nest observations. Thereafter, infants were capable of independent travel and nesting/parking ceased. During nest observations, a third observer used instantaneous focal nest scans conducted at 5‐min intervals to record patterns of nest use, reuse, and nest sharing, including the number and location of nests and park sites used, the relative proportions of time females spent in each nest, rates of nest transfer, and nesting strategy (single, communal, dual purpose nest, and park locations). At each sampling point, the observer recorded the Nest ID and GPS coordinates (see below), as well as the number and identity of litters in the nest. We measured initial litter size as the number of visible offspring counted in each female's natal nest. Living infants move around in the nest and are generally visible. This measure was used as a minimum estimate of litter size, as it did not include infants that may have been stillborn or that died prior to being counted. We monitored infant survival by counting the number of infants alive during each subsequent focal observation of the respective female. We also opportunistically monitored litter size changes for those females who were not the subjects of a given day's focal sampling. Infant survival in this study was monitoring until December, when infants were traveling with mothers independently and nesting/parking ceased.

#### 2.3.2 Geospatial and ecological data

During behavioral observations, GPS coordinates were collected at 10‐min intervals from as close to the focal individual as possible to document individual range use. All observed feeding trees were marked (with aluminum tags), georeferenced, and assigned unique identification numbers (Tree ID, *n* = 637). For each feeding tree, we also recorded its taxonomic assignment (vernacular, as well as Genus and species, whenever possible), diameter at breast height (DBH), and height (estimated in meters). Similarly, all observed nesting trees received a unique Nest ID (*n* = 40). In this study, nesting trees were defined as trees in which nest construction was *directly* observed. Because it is often difficult to discern even known nests from tree tangles and lianas, all other sites that were not observed in some stage of construction were referred to as “park sites” and assigned a unique Park ID (*n* = 171). Whenever a Nest ID/Park ID was first encountered, we collected its location (via GPS coordinates), taxonomic assignment (vernacular, as well as Genus and species, whenever possible), DBH, and whether the tree was a known *Varecia* feeding tree (i.e., whether it also had a Tree ID). We also estimated the height and diameter of the nest, its location in the tree (e.g., near trunk, terminal branches), and noted the builder's identity (Female ID) whenever possible. Because data on nest/park locations were collected opportunistically and were done during behavioral observations, detailed descriptions of nest/park sites (*n* = 211) were not always possible. Thus, not all variables described above were available for all nest/park sites.

To allow for statistical comparison, an equal number (*n* = 211) of non‐nesting/parking (control) trees were selected from throughout the subjects’ range, and tree characteristics were collected following the methods described above. Efforts were made to select trees that were representative of the distribution and diversity of trees found throughout each female's home range, as determined by the diversity and distribution of trees found within botanical plots located throughout the communal range (see Baden, 2011 for details).

Finally, we selected a subset of 20 nests for detailed microhabitat sampling. These nests were randomly selected from the 40 nests included in our study and were evenly distributed among mothers. For each nesting tree in the subset, we established a 10 × 10 m plot with the nesting tree at its center. For each 100‐m^2^ plot, we collected the following data: (1) altitude (m); (2) slope; (3) aspect; (4) percent (%), (5) height (m), and (6) type of ground cover (e.g., grasses, leaf litter); (7) % canopy cover; (8) number and (9) density of trees; (10) average tree DBH (cm), (11) height (m), and (12) crown diameter (cm); and (13) percentage and (14) type of water cover (e.g., streams, rivers). Variables were selected based on their relevance to nest choice in earlier vertebrate studies. Variables 1–3 measured aspects of topography. Variables 4–6 measured aspects of ground cover and were used to estimate a subject's ability to detect terrestrial predators from the nest. Variables 7–12 measured aspects of forest structure and were used to estimate a nest's protection from aerial predators and/or the elements, either by providing cover to or escape routes from the nest. Variables 13–14 measure the presence and size of water features (e.g., streams, riverbeds) and were used to estimate drowning hazards in the event that infants fell from their nests. We then used a randomly generated azimuth (0–359°) and distance (1–10 m) from the edge of each 100‐m^2^ nest plot to obtain a paired random control site. Control and nest plots never overlapped. Using the methods described above, we collected the same 14 variables representing available microhabitat within the area.

#### 2.3.3 Spatial analysis

Home range analyses were performed with home range tools (HRT; Rodgers, Carr, Beyer, Smith, & Kie, 2007) for ArcGIS (ESRI, Redlands, CA, USA). Kernel density estimates (KDEs) were used to calculate home ranges for each female using a bivariate normal distribution, rescaling X‐Y coordinates to unit variances as recommended by Silverman (1986). Raster cell size was set to 10 × 10 m to reflect the spatial resolution of the data. Home range size was evaluated using 95% kernel isopleths. Incremental area analysis was used to determine whether range areas reached asymptotes and were thus reliable estimates of home range size.

Kernel density estimates were combined with layers created from geospatially referenced nesting, parking, control, and feeding tree data, to create a map from which straight‐line Euclidean distances could be calculated between all pairs of nests, park sites, and georeferenced feeding and control trees, as well as counts of all known feeding trees within a 75 m radius for inclusion in later statistical analyses. A radius of 75 m was chosen over other distances because mothers typically fed within 75 m of the natal nest during the earliest stages of infant development (A. L. Baden, unpublished data), making this a biologically meaningful distance to a mother's nest site selection.

#### 2.3.4 Statistical analyses

Statistical analyses were conducted in R version 3.3.2 (R Core Team, 2013). To characterize patterns of nest construction and nest site selection, descriptive statistics were calculated including the number, structure, and habitat characteristics of the nests built, as well as details of nest use, reuse, and rates of nest transfer. Note that data on nest site characteristics and nesting behaviors derive from the 6‐month study period, whereas all feeding trees recorded during our 17‐month study (see above) were included as known feeding trees in our analyses.

A series of logistic regressions were used to investigate the construction and use of sites. First, a logistic regression was used to explore the environmental variables predicting the site of nest construction at a range‐wide scale, using a dataset that included all nest (*n* = 40) and control (*n* = 211) trees and included Build (Y/N) as the dependent variable. Fixed effects included tree DBH (cm), the number of feeding trees within a 75 m radius, the average distance to all feeding trees within a 75 m radius, and whether the nesting tree was either a species of feeding tree (Y/N) or a known feeding tree (Y/N) as fixed effects. In cases of missing data, means for that variable were imputed prior to model building. In cases where means could not be imputed (e.g., Species of feeding tree), that nest was excluded from analysis.

Next, I explored the best predictors of nest use (Use Y/N). This was again done using a logistic regression, though this time using a subset of the earlier Nest (Y/N) dataset that included only used (*n* = 15) and unused nests (*n* = 25). In this analysis, the dependent variable was nest use (Y/N). Fixed effects were the same as those described above.

Finally, because it was often difficult to discern nests from park sites, I chose to investigate nest site selection more broadly, this time using a larger dataset of used nest *and* park sites (*n* = 211) and control trees (*n* = 211). In this case, the dependent variable was again Use (Y/N), with the fixed effects including tree DBH (cm), the number of feeding trees within a 75 m radius, the average distance to all feeding trees within a 75 m radius, and whether the nesting tree was either a species of feeding tree (Y/N) or a known feeding tree (Y/N), as well as the average distance to a female's own nest and park locations, as well as the average distance to others’ nest and park locations.

Prior to logistic regressions, predictor variables were assessed for collinearity using variance inflation factors (VIFs) (R version 3.3.2, usdm package, Naimi, 2014). VIFs were low across predictor variables, and thus, all predictor variables were included in all analyses.

I assessed model performance using an adjusted measure of Akaike's information criterion (AIC_c_) with the “dredge” function in the MuMIn package (Barton, 2013). I evaluated models using the change in AICc scores (AIC_c_) and Akaike weight value (*w*). The “best model” was the model with the lowest AIC_c_ score. As is the convention, I considered models within two AIC_c_ scores to be equally good (reviewed in Symonds & Moussalli, 2011).

I used a standard model averaging technique to estimate the effect sizes and significance values for each relevant parameter. To estimate the relative effect sizes of each term that appeared in any of the top models, I averaged the models in each of the 95% confidence sets (i.e., ΔAIC_c_ < 10). Model averaging with this threshold of confidence provides an additional and conservative method of estimating the effects of a given predictor (Burnham & Anderson, 2002).

I used likelihood ratio tests to compare final models to a null model with no fixed effects, thus verifying the statistical significance of the final model; I expected significant differences.

In some cases, there were too few data points to justify the use of logistic regression models (e.g., nest construction at the microhabitat scale, nest reuse). In these cases, nonparametric Kruskal–Wallis and Wilcoxon rank‐sum statistics were used (Rmisc package, Hope, 2013). Multiple comparisons were adjusted using Holm–Bonferroni correction (Abdi, 2010).

## 3 RESULTS

### 3.1 Reproductive behavior

Mating was observed in two of the eight reproductive‐aged females within the community and was restricted to two consecutive days in early July (2, 3 July) (Table 1). One female (Red) mated with a single male on a single day, while the other female (Orange) mated repeatedly with four separate males spanning a 2‐day period. These same two females were first located with infants 102 and 109 days later, respectively. Parturition likely occurred during the night or early morning hours, as both females had been followed the preceding day and were without infants until groups were left at 18:00 h. Timing of mating was estimated for the remaining females in this study by counting back 102–109 days from when each was first found with infants (8–20 October; Table 1). From these estimates, mating spanned a maximum 2‐week period between 22 June and 7 July. No female in the community was observed mating after 3 July, despite contacting all females daily, suggesting the mating season was likely even more constrained than our 2‐week estimate.

**Table 1:** Reproductive parameters of black‐and‐white ruffed lemurs in Mangevo: timing of vaginal estrus, mating, and birth observed in five parous females

Female	Red	Orange	Yellow	Green	Blue
Seen in vaginal estrus	2 July	2,3 July	n.d.	n.d.	n.d.
Mated	2 July	2,3 July	28 Jun–7 July^a^	1–9 July^a^	25 Jun–1 July^a^
Mate(s)	rPS	RG, PO, YR, NC	n.d.	n.d.	n.d.
Pair demographics	Same core group	Multiple groups	n.d.	n.d.	n.d.
First located with infants	13‐Oct	20‐Oct	14‐Oct	16‐Oct	8 Oct
Date of parturition	13‐Oct	20‐Oct	11–14 Oct^b^	14–16 Oct^b^	8 Oct
Gestation length (days)	102	108–109	n.d.	n.d.	n.d.

Notes

n.d., no data.

^a^ Estimated using 102–109 day gestation period

^b^ Range of possible parturition dates; because females were not sampled daily, range consists of the number of days between observation bouts.

### 3.2 Nest construction

**Figure 1:**
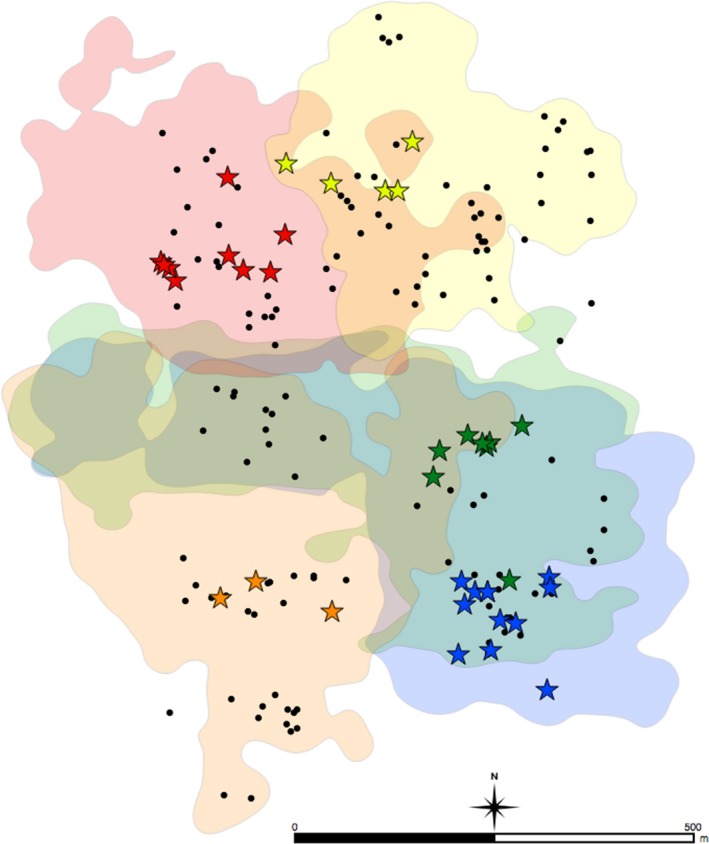
Map of individual annual female home ranges as calculated with 95% kernel density estimates from 17 months of ranging data (July 2007–December 2008) showing locations of all observed nest (stars) and park (circles) sites. Stars indicate known nesting localities (i.e., sites where nest construction was observed) and are color‐coded to reflect builder identity. Black circles indicate parking localities (i.e., sites where infants were left, but where no nest construction was observed)

Nest construction was first observed 37 days after mating and continued until parturition, when nest construction ceased. Females each constructed an average of 8.0 nests, though the number of nests constructed by females varied widely (range 3–15 nests) (Table 2). Nests were clustered in space within each female's home range (Figure 1), and individual female nests were separated, on average, by approximately 200 m (range = 13.1–746.0 m; Table 2). Females typically constructed their own nests away from other females’ nests at an average distance of 398.96 m (range = 287.6–956.6 m) (Table 2).

**Table 2:** Description of nest characteristics including total number of nests constructed, duration of nest construction, descriptions of nesting sites, and details of nest use

Female	*n*	Red	Orange	Yellow	Green	Blue	Mean	*SD*
Total number of nests constructed^a^	40	8	3	5	9	15	8.00	4.58
Total number of nests used (all)	40	3	3	2	5	2	3.00	1.22
Total number of nests used (own)	40	2	3	2	3	2	2.40	0.55
Total number of nests used (others)	40	1	0	0	2	0	0.60	0.89
Avg. time spent in nest construction (min:s)	19	8:48	*n*.d.	4:52	8:00	12:19	8:35	7:34
Avg. tree DBH (cm)	29	55.28	43.73	69.83	46.34	49.78	52.26	19.70
Avg. height in tree (m)	28	23.50	23.00	20.00	23.43	19.80	21.61	4.17
Avg. nest diameter (m)	16	0.92	1.25	1.25	1.20	1.75	1.22	0.74
N species used for nests	28	2.00	3.00	4.00	6.00	5.00	4.00	1.58
N nests constructed in a species of feeding tree	28	4.00	3.00	2.00	6.00	8.00	4.60	2.41
N nests constructed in a known feeding tree	28	0.00	0.00	0.00	0.00	0.00	0.00	0.00
Avg. distance to nests (own) (m)	37	137.69	91.90	85.78	248.12	277.51	199.96	194.52
Avg. distance to nests (others) (m)	37	400.46	376.75	413.90	319.16	446.65	398.96	146.06
Avg. density of feeding trees (n per 75 m)	37	32.75	14.00	22.60	24.25	21.46	23.01	8.35
Avg. distance to feeding trees (within 75 m)	37	51.22	51.64	47.19	45.56	51.14	49.36	3.29

^a^ “Nest sites” are those that were observed in some stage of nest construction; sites that were later used, but which females were not observed building are classified as “park sites”.

Gestating females were their nest's sole constructors; females were never observed participating in communal or coordinated nest construction, nor did males or nulliparous females (*n* = 1) exhibit nest construction behaviors. Of the 19 nest construction events for which detailed behavioral data were available, nest construction occurred most often following feeding bouts (10 of 19 recorded cases of nest construction, 52.6%), though it did also occur just after resting (eight of 19 cases, 42.1%) and self‐grooming events (two of 18 cases, 10.5%). Only one‐third of nest building events occurred in the vicinity of the builder's original activity (six of 19 cases); rather, a majority of nest construction events (66.7%) were immediately preceded by travel, after which time nest construction began.

When observed, nest construction took on a familiar form. During species‐typical behaviors (e.g., feeding, resting, self‐grooming, as described above), females suddenly began vocalizing, making low‐frequency, “growls” (sensu Pereira, Seeligson, & Macedonia, 1988) as they traveled through the trees. Although previously described as being short in duration (Pereira et al., 1988), growls observed in the nesting context differed in that they were longer, occurred in quick succession, and lasted the entirety of nest building behavior. Growls of this nature have only ever been observed in association with nest construction and infant care in this population.

Nest construction events began with females moving deliberately and quickly through the canopy and were virtually indistinguishable from foraging, except that it was accompanied by growl vocalizations, as described above. Upon locating nesting materials—typically a branch, which was often within close radius to the nest construction site (typically within 15–20 m)—females would chew‐off a piece of the branch, and orally carry the nesting material to the site of nest construction. In one case, a female dropped a branch during nest construction. This branch measured 62 cm in length (Supporting Information Figure S1). In all cases, nesting materials were placed among branches, lianas, and/or pre‐existing nesting materials using the “fetch and drop” method (sensu Hansell, 2005); branches were never woven together, and nests were almost always constructed with materials collected from within the same nesting tree. Nests resembled shallow bowls or platforms, but were never enclosed.

Nesting bouts were typically brief (range =1 min 22 s – 28 min; Table 2). Once nest construction ceased, females either resumed species‐typical behaviors in proximity to the nesting site (10 of 19 recorded construction events; 52.6%) or immediately traveled away from the site of nest construction and resumed normal activity elsewhere (47.7%). One‐third (33.3%) of nest building observations that were immediately preceded by travel were also followed by travel, suggesting that females may have visited the site explicitly for the purpose of nest construction.

Females returned to nest construction sites throughout gestation, periodically adding branches to pre‐existing nest locations, although detailed data on the total investment in individual nesting sites are unavailable. In some cases, nests were large and easily detected from the ground, while others were only identified as nests because of observed nest construction activities, therefore making actual constructed nests difficult to discern from later parking locations. Thus, only *known* nest sites (i.e., those which were observed during some stage of nest construction activity) are referred to as “nests”; all other locations are referred to as “park sites” from this point forward.

### 3.3 Nest site characteristics and selection

**Figure 2:**
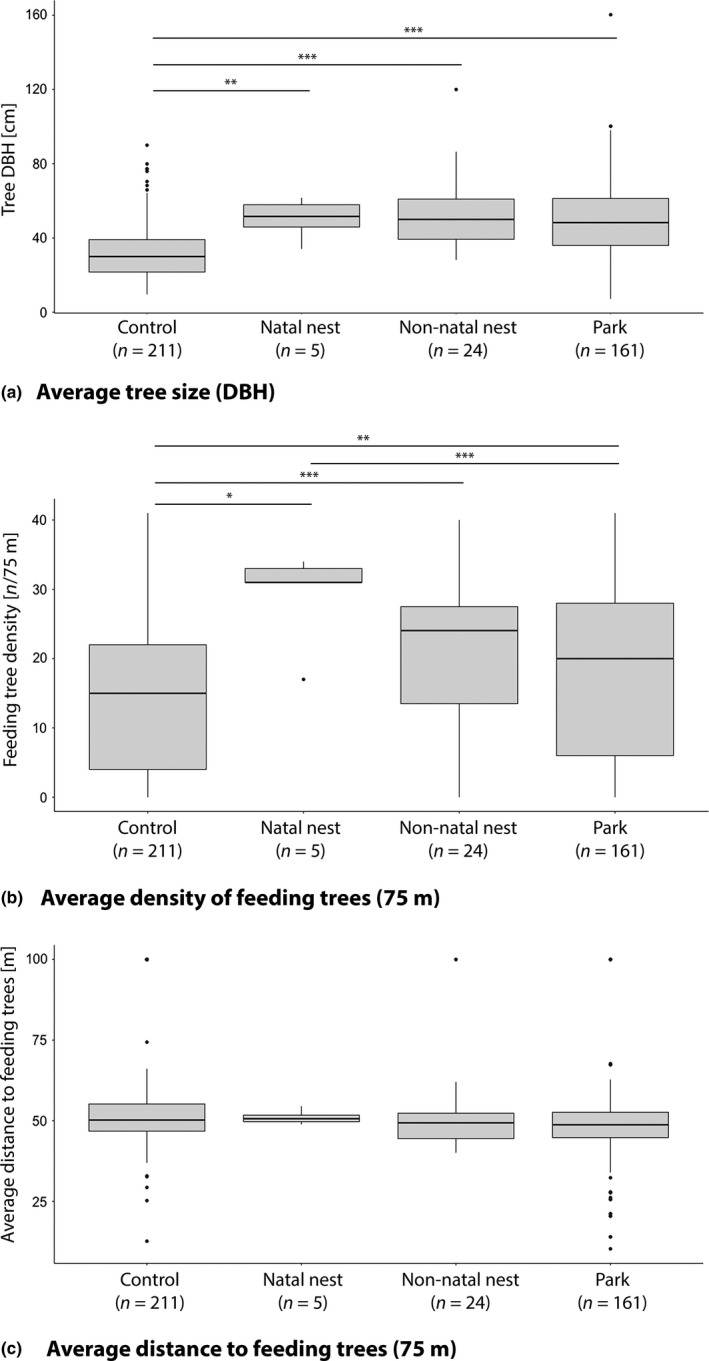
Box plots comparing (a) average tree size (diameter at breast height, DBH), (b) average density of feeding trees within 75 m, and (c) average distance to feeding trees within 75 m of nest, park, and control sites. Asterisks indicate significant pairwise comparisons as determined by post hoc Wilcoxon rank‐sum statistics with Holm–Bonferroni corrections. ***p < 0.001, **p < 0.01, *p < 0.05.

Nests averaged approximately 1.2 m in diameter (±0.74 *SD*, range = 0.5–2.5) and were built in the crux of branches (near the trunk of the tree) 21.61 m in the canopy (range = 15.0–32.0 m; Table 2). Nesting trees averaged 52.26 cm DBH (range = 28.20–120.00 cm; Table 2) and were significantly larger than control trees (*W* = 1,343.5, *p*‐value < 0.001; Figure 2).

Females constructed their nests in an average of four tree species (range = 2–6; Table 2), totaling fifteen tree species (Supporting Information Table S1) and representing only 4.34% of the total tree diversity currently recognized in Ranomafana (P.C. Wright, unpublished data). Ten of the 15 species used (66.7%) were preferred food species; however, subjects were never observed feeding in nesting trees prior to, during, or following the nesting season. That is, nesting trees were never *known* feeding trees (i.e., nests were never located in feeding trees that had been exploited during behavioral observations from our 17‐month study; 0 of 40 cases). Nesting trees were, however, located in areas with significantly higher densities of known feeding trees than control sites (*W* = 2,179.5, *p* < 0.001), though they did not differ significantly from control trees in average distance to said feeding trees within the range (*W* = 4,461.5, *p* > 0.05; Figure 2).

The best model predicting nest construction included the fixed effects of tree DBH, the number of known feeding trees within 75 m of the nesting site, and whether the nesting tree was itself a known feeding tree (Table 3). This model performed significantly better than the null model (χ^2^(3) = 120.12, *p* < 0.001). There was one other best model within two AIC_c_ scores that included all four predictor variables (DBH, N known feeding trees within 75 m, average distance to known feeding trees within 75 m, and whether the nest site was also a known feeding tree; Table 4). Nests were significantly more likely to be built in large trees (DBH) situated in stands with a high density of *known* feeding trees (i.e., trees that are currently or have previously been exploited; Table 3). The best model also indicated that known feeding trees were less likely to serve as nesting sites, though this variable was not a significant predictor of nest site selection in the model.

**Table 3:** The best model resulting from a logistic regression predicting nest construction

Fixed factor	Estimate	*SE*	Adjusted *SE*	*t*	*p*‐Value
(Intercept)	−4.96	1.2	1.2	4.02	0.00
**TreeDBH**	**0.08**	**0.0**	**0.0**	**4.02**	**0.00**
**FDTree_Count_75m**	**0.07**	**0.0**	**0.0**	**2.93**	**0.00**
FDTree_KnownYES	−20.12	1,282.7	1,289.1	0.02	0.99
FDTree_Dist_75m	−0.01	0.0	0.0	0.56	0.58

Notes

Data included all nest (*n* = 40) and control (*n* = 211) trees and used Build (Y/N) as the dependent variable. Fixed effects included tree DBH (TreeDBH), the number of feeding trees within a 75 m radius (FDTree_Count_75,m), the average distance to all feeding trees within a 75 m radius (FDTree_Dist_75m), and whether the nesting tree was either a species of feeding tree (FDTree_Species) or a known feeding tree (FDTree_Known) as fixed effects. This model performed significantly better than the null model (χ^2^(3) = 120.12, *p* < 0.001). Values presented in bold are significant at *p* < 0.05.

**Table 4:** Top 10 models of fixed effects on the nest construction as determined by the number of (N FD Trees) and distance to feeding trees within 75 m (Distance to FD Trees), tree DBH (DBH), and whether the nest site was located in a known feeding tree (Known FD Tree)

Model	Fixed factors	*df*	logLik	AIC_c_	∆AIC_c_	Weight
**1**	**N FD Trees** + **Known FD Tree** + **DBH**	**4**	−**50.03**	**108.23**	**0.00**	**0.69**
**2**	**N FD Trees** + **Distance to FD Trees** + **Known FD Tree** + **DBH**	**5**	−**49.91**	**110.06**	**1.83**	**0.28**
3	Distance to FD Trees + Known FD Tree + DBH	4	−53.56	115.29	7.06	0.02
4	Known FD Tree + DBH	3	−55.71	117.52	9.29	0.01
5	N FD Trees + Known FD Tree	3	−62.25	130.59	22.36	0
6	N FD Trees + Distance to FD Trees + Known FD Tree	4	−62.21	132.59	24.36	0
7	Distance to FD Trees + Known FD Tree	3	−69.46	145.01	36.78	0
8	Known FD Tree	2	−70.72	145.49	37.27	0
9	N FD Trees + DBH	3	−80.48	167.05	58.82	0
10	N FD Trees + Distance to FD Trees + DBH	4	−80.33	168.81	60.58	0

Notes

Models in bold are within 2 AICc scores of the best model and are considered equally good.

A series of nonparametric Wilcoxon rank‐sum tests with Holm–Bonferroni correction were used to further elucidate the microhabitat characteristics related to nest site preference (i.e., nest site vs. control site). Of these, only DBH was significant at the *p* < 0.05‐level (*W* = 119, *p* = 0.048). Percent crown cover also approached significance (*p* = 0.088; Supporting Information Table S2), although neither was significant after Holm–Bonferroni corrections. All other microhabitat characteristics, including the altitude, slope, aspect, ground cover and average size and density of all trees within the 10 × 10 m plots, did not differ significantly between control and nesting sites (Supporting Information Table S2). Nests were never built in areas with water cover (i.e., never located over rivers or streams), although water cover was also absent from all control sites.

### 3.4 Nest use

Females used only a fraction of their constructed nests for birth and infant rearing. On average, each female used a total of 3.0 nests (range, 2–5; Table 2). Using subsampled data that included nests only, the best model predicting nest use included the size (DBH) of the nesting tree and the average distance between the nesting tree and nearby feeding trees, though these variables only approached significance (Table 5A). There were no other best models within 2 AIC_c_ scores (Table 6), and the best model significantly outperformed the null χ^2^(37) = 41.397, *p* = 0.009.

**Table 5:** The best models predicting nest use (A) and nest and park site use (B) resulting from logistic regressions

	Estimate	*SE*	Adjusted *SE*	*t*	*p*‐Value
A. Nests only					
(Intercept)	−0.58263	2.90194	2.97199	0.196	0.845
*FDTree_Dist_75m*	*0.08478*	*0.04585*	*0.04735*	*1.791*	*0.073*
*Tree.DBH*	−*0.0852*	*0.04431*	*0.04569*	*1.865*	*0.062*
FDTree_Count_75m	−0.02447	0.04686	0.04818	0.508	0.612
B. Nests and parks					
(Intercept)	−1.7583861	0.562608	0.5636551	3.12	0.002
**FDTree_Dist_75m**	−**0.0153822**	**0.0074284**	**0.0074499**	**2.065**	**0.039**
**Tree.DBH**	**0.050503**	**0.0067541**	**0.0067737**	**7.456**	**<0.001**
FDTree_Count_75m	−0.0002631	0.0098422	0.0098694	0.027	0.979

Notes

Nest use was best predicted by the size of the nesting tree (TreeDBH) and the average distance between the nesting tree and nearby feeding trees (FDTree_Dist_75m), though these variables only approached significance. This model outperformed the null χ^2^(37) =41.397, *p* = 0.009. The use of nest and park sites was best predicted by tree size (TreeDBH) and average distance to nearby feeding trees (FDTree_Dist_75m). The best model significantly outperformed the null (χ^2^(419)=499.05, *p* = 0.033). Values presented in bold are significant at *p* < 0.05. Values presented in italics are significant at *p* < 0.10.

**Table 6:** Top eight models of fixed effects on nest use (A) and nest and park site use (B) as determined by the number of (N FD Trees) and distance to feeding trees within 75 m (Distance to FD Trees), tree DBH (DBH), and whether the nest site was located in a known feeding tree (Known FD Tree)

Model	Fixed factors	*df*	logLik	AIC_c_	ΔAIC_c_	Weight
A. Nests only						
**1**	**Distance to FD Trees** + **DBH**	**3**	−**20.7**	**48.06**	**0**	**0.56**
2	N FD Trees + Distance to FD Trees + DBH	4	−20.68	50.5	2.43	0.17
3	Distance to FD Trees + DBH	2	−23.86	52.05	3.99	0.08
4	N FD Trees + DBH	3	−22.94	52.54	4.48	0.06
5	DBH	2	−24.16	52.64	4.58	0.06
6	(Null)	1	−25.9	53.9	5.84	0.03
7	N FD Trees + Distance to FD Trees	3	−23.7	54.06	6	0.03
8	N FD Trees	2	−24.93	54.19	6.12	0.03
B. Nests and parks						
**1**	**Distance to FD Trees** + **DBH**	**3**	−**249.5**	**505.1**	**0.00**	**0.57**
2	N FD Trees + Distance to FD Trees + DBH	4	−249.5	507.1	2.02	0.21
3	DBH	2	−251.8	507.7	2.54	0.16
4	N FD Trees + DBH	3	−251.7	509.5	4.42	0.06
5	N FD Trees + Distance to FD Trees	3	−285.4	576.8	71.65	0.00
6	Distance to FD Trees	2	−286.8	577.6	72.45	0.00
7	N FD Trees	2	−286.9	577.7	72.62	0.00
8	(Null)	1	−289.3	580.6	75.49	0.00

Notes

The model in bold is the only best‐performing model. No other model is within 2 AICc scores.

Among the nests chosen, each female chose a “natal nest” (*sensu* Baden et al., 2013), which was used for infant birth and the earliest stages of infant development. Trees used for natal nesting did not differ significantly in size from the non‐natal nests and parking locations females later used (Figure 2). However, natal and non‐natal nests and park trees were all significantly larger than control trees, both combined (*W* = 22,268, *p* < 0.001) and individually (Figure 2a). Although small sample size (n = 5) precluded model‐building, nest and park sites were also located in areas of significantly higher feeding tree density than were control sites (Figure 2b). Average distance to nearby feeding trees did not differ significantly between nest, park, or control sites (Figure 2c).

Females kept infants exclusively in natal nests for an average of 13.8 days after birth (±8.47 *SD*, range = 3–22), after which time females began to transfer infants regularly between non‐natal nests. By approximately 3.4 weeks of infant age (±0.89 *SD*, range = 2–4 weeks), females also began parking infants in trees without nesting structures (i.e., park sites). Even after the onset of parking, nests were used periodically throughout infant development until nesting/parking ceased.

**Figure 3:**
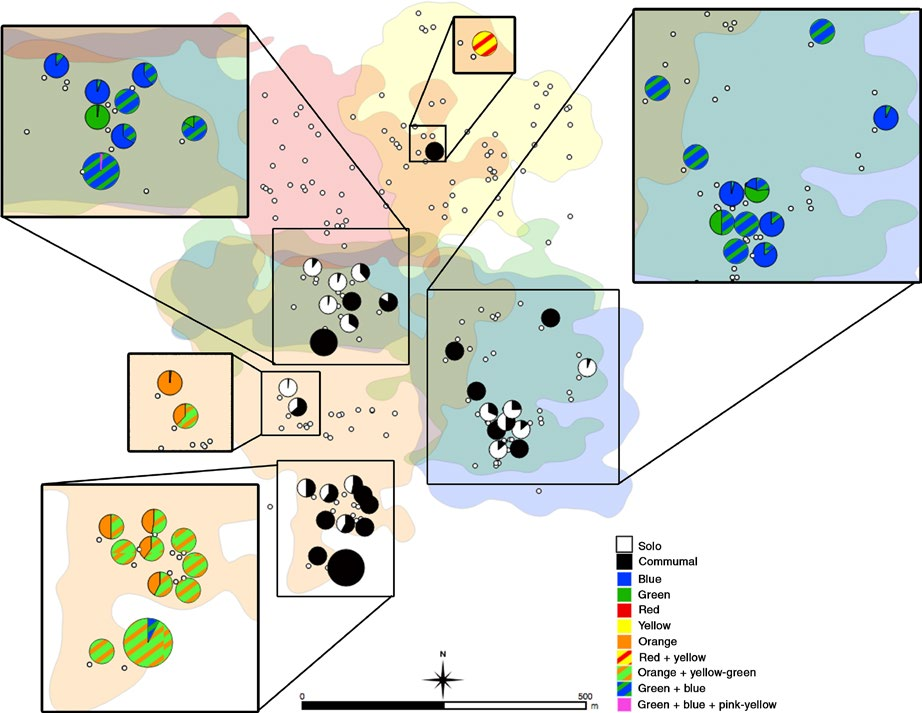
Map illustrating the relative time nests and park localities were used singly (solo) versus communally (communal), as well as the identities of site users. Points indicate nest and park sites. Point size indicates the total number of users (range: 1, smallest–4, largest). Point color on the main map differentiates solo (white) from communally (black) used sites. Pie charts on the main map represent sites used by more than one individual and illustrate the proportion of time each site was used for solo (white) versus communal nesting and parking (black). Pie charts in inset maps illustrate the identities of site users, as well as the proportion of time each female (or females) used dual‐use and/or communal use sites. Solid colors indicate solo user identity; hashed lines indicate identities of communal nesters (i.e., simultaneous nest use). Striped regions of the pie charts indicate communal use by two females simultaneously. Note that females Yellow–Green and Pink–Yellow were peripheral females and were only followed opportunistically.

Of the 40 nests included in this study, 62.5% (25/40) were abandoned (i.e., never used), 37.5% (15/40) were used singly (i.e., used by one female at a time), and 2.5% (1/40) were used communally (i.e., used by ≥2 females simultaneously; Figure 3). Of these, one nest (305) was used both singly (by female Green) and communally (by females Green and Blue simultaneously) on separate occasions.

In contrast to the limited number of nests used by females, parking sites were far more plentiful (mean = 31.4 park sites ±9.10 *SD*, range = 23–47; Table 7). Unlike nesting trees, parking sites were sometimes located in known feeding trees, although still only occasionally (8.7%; Table 7). With the addition of park sites, the combined use of nest and park sites was best predicted by tree size (DBH) and average distance to nearby feeding trees (Table 5B). There were no other best models, and the best model significantly outperformed the null (χ^2^(419) = 499.05, *p* = 0.033; Table 6).

**Table 7:** Description of park site characteristics including total number of park locations and specifics of parking sites

Female	Red	Orange	Yellow	Green	Blue	Avg.	*SD*
Total number of park locations used[Link]	23	29	47	29	29	31.40	9.10
Number of park trees also used for feeding	3	1	4	3	2	2.60	1.14
Avg. tree DBH (cm)	54.56	46.80	47.20	59.85	61.30	51.61	20.65
Avg. height in tree (m)	22.91	23.90	23.10	26.50	24.70	24.25	5.94
Avg. distance to park sites (own) (m)	166.08	128.80	230.20	263.66	263.88	214.14	136.43
Avg. distance to park sites (other) (m)	402.99	391.08	428.26	351.52	340.33	390.85	100.00
Avg. density of feeding trees (*n* per 75 m)	28.96	8.20	14.50	24.93	25.24	18.13	11.86
Avg. distance to feeding tree (within 75 m)[Link]	50.08	43.40	47.80	48.64	47.88	46.76	9.38

Park locations exclude confirmed nest locations (those observed in some stage of construction).

Distance calculated as average distance within a 75 m radius of the park site.

In contrast to nest sites, park sites were more commonly used for infant crèching. The majority of park sites were used singly (69.4%, 109/157); 30.6% (48/157) of park sites were used communally. Of those, 8.3% (13/157) were used for single and communal nesting events on different occasions. Most solo parking sites were only ever used by a single female (*n* = 107 of 109 solo park sites); in some cases, however, solo parking sites were used by two females on separate occasions (*n* = 2 of 109 park sites). In both cases, these sites were used by females Green and Blue (Figure 3). Communal park sites were used by as many as four females (average = 1.91 females ± 0.73 *SD*), although no more than two litters were crèched in a single park site at any one time (Figure 3).

### 3.5 Nest reuse

Females routinely reused nests and park sites, though *natal* nest locations were abandoned after initial nest transfer. Nesting and parking sites were reused and were used in both single and communal nesting contexts (Figure 3). Nesting/parking sites were reused an average of 1.90 times (±1.18 *SD*, range = 1–5) before being abandoned. Reused nest/park sites did not differ significantly from sites that were used only once, although there was a trend toward reused sites being in closer proximity to feeding trees within 75 m than single‐use sites (*W* = 1634.5, *p* = 0.073). In some cases, reuse occurred within a single day, while in other cases, nests and park locations were reused as many as five times across several months. In most cases, sites were reused by a single individual, though in nine instances, sites were reused by two subjects simultaneously (either Blue and Green or Orange and Yellow‐Green).

### 3.6 Nest site selection and reproductive success

Infant survival was unrelated to many aspects of nest site selection, including the average DBH of nest/park trees a female used (*r*_s_ = 0.564, *p* = 0.322), the average density of feeding trees in which nest/park sites were situated (*r*_s_ = −0.154, *p* = 0.805), or the average distance of a female's nest/park sites to feeding trees (*r*_s_ = −0.410, *p* = 0.493) or to her own nest/park sites in the area (*r*_s_ = 0.667, *p* = 0.219). The relationship between infant survival and a female's average proximity to *others’* nest/park sites did, however, approach significance (*r*_s_ = −0.872, *p* = 0.054), such that females who used nest/park sites that were in closer proximity to their neighbors’ tended to have higher infant survival than did females whose nests and park sites were more isolated.

## 4 DISCUSSION

### 4.1 Nest site selection

In this study, I describe nest construction by five ruffed lemur females during the only recorded reproductive event in 6 years of observation. All focal females built at least three nests, several of which were used for birth and throughout early infant development. An additional two females bred within the community during this same season, but were only followed opportunistically when their infants were crèched together with those of focal females. Although nest construction was only observed for five of the seven females included in this study, crèching sometimes occurred in nests (i.e., sites where nest structures clearly existed) whose builder was not known. In these cases, nests were classified as “park sites” in analyses to conform with our definitions. While it is possible that nests were built by focal females on days when they were not the subject of focal observations, it is equally likely that nonfocal females (e.g., Green‐Yellow, Pink‐Yellow) constructed the nests in question. It is therefore likely that all breeding females within the community constructed nests for reproduction, despite not being observed doing so. Thus, it is expected that, unlike communal nesting, which is facultative in the species (Baden et al., 2013), nest construction is obligate and ubiquitous in ruffed lemurs.

Nesting structures described herein differ from those described in earlier studies. Nests observed in the Mangevo community were moderately sized, simple platform or shallow bowl‐shaped structures; nests were never enclosed, as described by Vasey (2007). Nest construction lasted over a three‐month period (gestation) using the “fetch and drop” method, whereby a structure is built by “simple, repeated elements of behavior via the accumulation of objects deposited at the same location or in particular location to one another” (Hansell, 2005). This is in contrast to earlier accounts of ruffed lemur nesting behaviors, which described females as weaving branches and lianas together to form more intricate, structurally sound nests (P.C. Wright, personal communication). Whether these discrepancies are due to regional, temporal, or “cultural” variation in nesting behaviors, or simply a consequence of small sample size in existing studies is impossible to address at this time. However, research into ruffed lemur reproduction and nesting behaviors is ongoing at both Andranobe and Mangevo sites, and will allow a more robust analysis of these and other comparisons in future studies.

Females preferentially built nests in large trees (i.e., those with large basal diameter) belonging to only fifteen species, a fraction of the overall tree diversity found at the site. These fifteen species comprised among the largest trees present in the forest at the time of this study (Baden, 2011) and were likely chosen for their ability to safely support nesting structures for mothers and their large litters of underdeveloped young. Nests were frequently built in species of feeding trees, though never in *known* feeding trees; that is, females were never observed feeding in trees where nests were located. Nests were, however, located in stands with a relatively high density of known feeding trees nearby. In combination, these results suggest that nest site selection in black‐and‐white ruffed lemurs was driven primarily by the need to provide structural support for nests, and access to high‐quality food resources for lactating mothers. These results are in concordance with patterns of nest site selection observed in several other litter‐bearing mammals, including Arizona gray squirrels, whose preference for large trees with extensive crowns in areas of high tree density increases access to food and minimizes travel distances to food sources (Cudworth & Koprowski, 2011); fat dormice, who prefer to nest in dense forest stands with high numbers of oak trees, the acorns of which are an important food source (Juškaitis & Šiožinytė, 2008); and hazel dormice, who seek sites that guarantee a continuous food supply in the vicinity of nests (Juškaitis et al., 2013).

Nest sites in the present study did not differ significantly from controls in aspects of their microhabitat, including canopy, ground, and/or water cover and surrounding tree size and density (i.e., considering *all* trees within a 10 × 10 m plot), characteristics that were predicted to provide protection from the elements and/or predators, either by shielding nests from rain and/or concealing nests against aerial predators (high % canopy cover), or by allowing the detection (low % ground cover) and avoidance of terrestrial predators (high tree density or size) by increasing visibility and providing multiple escape routes. These results corroborate earlier studies that have found the thermoregulatory and antipredator benefits of nest use to be secondary (birds: Heenan & Seymour, 2011; primates: Hediger, 1977; Kappeler, 1998) or unimportant to nesting decisions (Heenan & Seymour, 2011; Tomás et al., 2006).

Alternatively, it is possible that the microhabitat variables from this study were insufficient to allow us to test these hypotheses, either in that sample size was too small or that measurements were simply collected at the wrong spatial scale. Recent work has found that different predictors may matter at different spatial scales. For example, in a study of bonobo nest site selection, forest structure, availability of fruit trees, and terrestrial herbaceous vegetation were important predictors of nest site selection at 750 m, <600 m, and <300 m, respectively (Serckx et al., 2016). Thus, future studies of nest site selection in ruffed lemurs would benefit from a systematic consideration of potentially relevant variables at increasing spatial scales to assess their value to nesting decisions.

Previous studies have found that social cues may also be important predictors of nest site selection and use (e.g., Pike, Webb, & Andrews, 2011; Loukola et al., 2012; Kivelä et al., 2014). In the current study, however, social cues (distance to other females’ nests + park sites) were poor predictors of nest use. This was surprising, particularly because proximity to conspecifics’ nest/park localities was the only variable related to infant survival that approached significance. One possibility is that the structure and location of nests were less important to infant survival than were strategies of nest *use*. In this study, there was a trend toward increased infant survival in females that used nests located in closer proximity to those of their social partners. This result aligns with previous findings, where the presence and intensity of crèching (i.e., communal nesting) were strongly related to infant survival, such that infants who were communally nested for longer periods of time experienced significantly higher survival than did infants who were singly nested or crèched less often (Baden et al., 2013). Taken together, these lines of evidence suggest that patterns of female nest site selection and use may set the stage for communal crèching during periods of facultative allomaternal care in this species.

Alternatively, it is possible that social cues are more important on a longer timescale and that we simply have not amassed the long‐term data necessary to test this hypothesis. Collared flycatchers, for example, use conspecific cues with a time lag of 1 year (Kivelä et al., 2014). The birds preferred nest sites that had been previously occupied, or that were in proximity to nests where conspecifics had high breeding success in previous years, and for locales that were surrounded by active nests. Unfortunately, due to the infrequent and unpredictable nature of ruffed lemur reproduction (Baden et al., 2013; Vasey, 2007), I was unable to consider long‐term social variables in my analyses. Future work will consider whether nest site characteristics, allomaternal help, or a combination of the two contribute to the survival of infants and lifetime reproductive success of mothers.

### 4.2 Multiple nest building and nest use

In most studies, variables predicting nest site selection and use are one and the same. This is because most taxa invest in a single nest per breeding attempt. Some species, including ruffed lemurs, however, simultaneously build multiple nests in anticipation of birth (Beckmann & Martin, 2016; Berg et al., 2006; Sumasgutner, Millán, Curtis, Koelsag, & Amar, 2016). There is evidence that both the selection of a nest site and the quality of a nest can have important effects on breeding success (Hoi, Schleicher, & Valera, 1994, 1996; Thompson & Furness, 1991; Weidinger, 2002), and many species expend considerable time and energy in the construction of nests for breeding (Collias & Collias, 1984; Metz, 1991; Verner & Engelsen, 1970). Given the large energy costs involved in nest building, the reason for multiple nest building—particularly those that are not ultimately used—is thus often unclear.

Several adaptive hypotheses for multiple nest building have been proposed. Early studies suggested that multiple nests are built for practice (Hunter, 1900), to demarcate territory boundaries (Allen, 1923), and to expend excess energy (Forbush, 1929). While these have received little support, several other hypotheses are more tenable. For example, there has been some support for hypotheses suggesting that multiple nest building is related to mate attraction (Evans & Burn, 1996; Garson, 1980), anti‐predation strategies (e.g., decoys: Watts, 1987), coping with nest competition (Sumasgutner et al., 2016), and/or coping with the destruction of nests during storm events or other natural disturbances (Elkins, 2010). Of these, the most common hypothesis is that nests are used in sexual selection, where individuals (typically males) build multiple nests to signal the quality of the nest building individual (Evans & Burn, 1996; Garson, 1980), or perhaps to signal the quality of the territory (Evans, 1997). In this study, I found that ruffed lemur nest building occurs after mating, and is performed only by females. Sexual selection therefore cannot explain multiple nest building as a means of attracting mates, as mate selection occurs prior to the onset of nest construction events. Moreover, this species’ behavioral ecology is such that nest building cannot be easily explained by territoriality or nest competition, as the subjects of this study were members of a single behavioral community that regularly participated in communal infant care, including infant crèching and nest sharing (Baden et al., 2013; Baden et al., 2016; this study). And while natural disturbances are possible drivers of nest abandonment, we witnessed females returning to several nests repeatedly throughout gestation, suggesting that disturbance was not a primary driver of these behaviors.

Of the hypotheses used to explain multiple nest building behaviors in other vertebrates, only the anti‐predator response poses a possible and intriguing argument. Birds will often build multiple nests in high predator density areas and/or abandon nests prior to use if disturbed during early stages of construction (e.g., Berger‐tal, Berger‐tal, & Munro, 2010; Flegeltaub et al., 2017). While we did not witness nest disturbance, fossa predation in the Mangevo community was high during the gestation period. In 2008, five individuals from a neighboring community fell victim to predation events within a single month (A. L. Baden, unpublished data). Thus, it is possible and even likely that multiple nests were built to reduce the probability of predation events, although the actual mechanism by which they would function is unclear. Multiple nests have been hypothesized to serve several anti‐predator functions. Some have hypothesized that multiple nests may serve as decoys, whereby “extra” nests may distract predators from breeding nests and decrease the probability that a predator will discover an active nest (Berg et al., 2006; Flegeltaub et al., 2017; Leonard & Picman, 1987; Watts, 1987). Alternatively, multiple nest building may be a strategy to avoid predator attraction either by allowing females to regularly transfer infants among nests (e.g., because feces may attract predators by its odor or appearance, Petit et al. 1989; but see Soanes, Peters, Delhey, & Doody, 2015) or as way to allow mothers to adjust nest use and opportunistically avoid certain nests altogether (e.g., due to the unexpected arrival of a predator to the area or a shift in the dominant predator type, as described by Beckmann and Martin (2016). Unfortunately, the predator avoidance hypothesis is also among the most difficult to test because predation events are rare and difficult to observe (Stanford, 2002) and experimentally manipulating nesting behaviors is ill‐advised in a Critically Endangered species with a slow, unpredictable breeding pattern (Baden et al., 2013). In future studies, it would be interesting to compare patterns of nest building during years with and without predation events (e.g., whether females vacillate between multiple and single nest construction in times with and without predator threat, respectively) to further test whether this hypothesis garners support.

Based on the results of this study, I propose yet another hypothesis to explain multiple nest building in ruffed lemurs: that multiple nest building is used to facilitate access to reliable, high abundance, high‐quality food resources in a litter‐bearing primate with prolonged infant dependence. Under this scenario, gestating females seek out large trees (i.e., those of large basal diameter likely to provide ample support and stability to nests housing altricial young) located in areas of high feeding tree density in which to construct their nests. Because nests are built as many as three months before the birth season begins, it is possible that females are constructing nests in areas where the probability of fruit availability in coming months is highest. Rather than responding to current resource availability, it seems likely that nest construction is done in anticipation of future resource potential and that decisions during nest *use* are based on the actual/realized phenological patterns at that time. To test this hypothesis would require monitoring the phenological stage of feeding trees in proximity to all nest and control sites throughout gestation and lactation to determine whether nest *use* is related to the availability, abundance, and perhaps quality of resources nearby, and whether abandoned or unused nests are simply located in areas of relatively lower productivity. Of course, it is always possible that nests classified as “unused” in our study were actually used by subjects on days when they were not being followed (subjects were typically followed every other day). Thus, future research would benefit from additional observers and/or long‐term research to test whether these same patterns hold. Finally, I hypothesize that the feeding benefit provided by nests in proximity to feeding trees does not, in itself, significantly increase infant survival, but rather only in combination with communal nesting behaviors.

In conclusion, results from this study suggest a complex pattern of nesting behaviors that involves females strategically building nests in areas with high potential resource abundance, using nests in areas according to their realized productivity, and communally rearing infants within a network of nests distributed throughout the larger communal territory. Whether and how this strategy varies regionally, temporally, or “culturally” remains to be addressed. Testing these hypotheses and others will require longitudinal studies and, of course, additional reproductive events.

## CONFLICT OF INTEREST

None declared.

## AUTHOR CONTRIBUTIONS

ALB conceived of the project, collected and analyzed the data, and wrote the article.

## Data Availability

Data can be accessed on the Dryad repository (https://doi.org/10.5061/dryad.7n6f407).
