# Supplementary figures and images for: A description of nesting behaviors, including factors impacting nest site selection, in black‐and‐white ruffed lemurs (Varecia variegata)
Source: Ecol Evol. 2019 Jan 1;9(3):1010–28. doi: 10.1002/ece3.4735 (PMC6374655; doi:10.1002/ece3.4735)

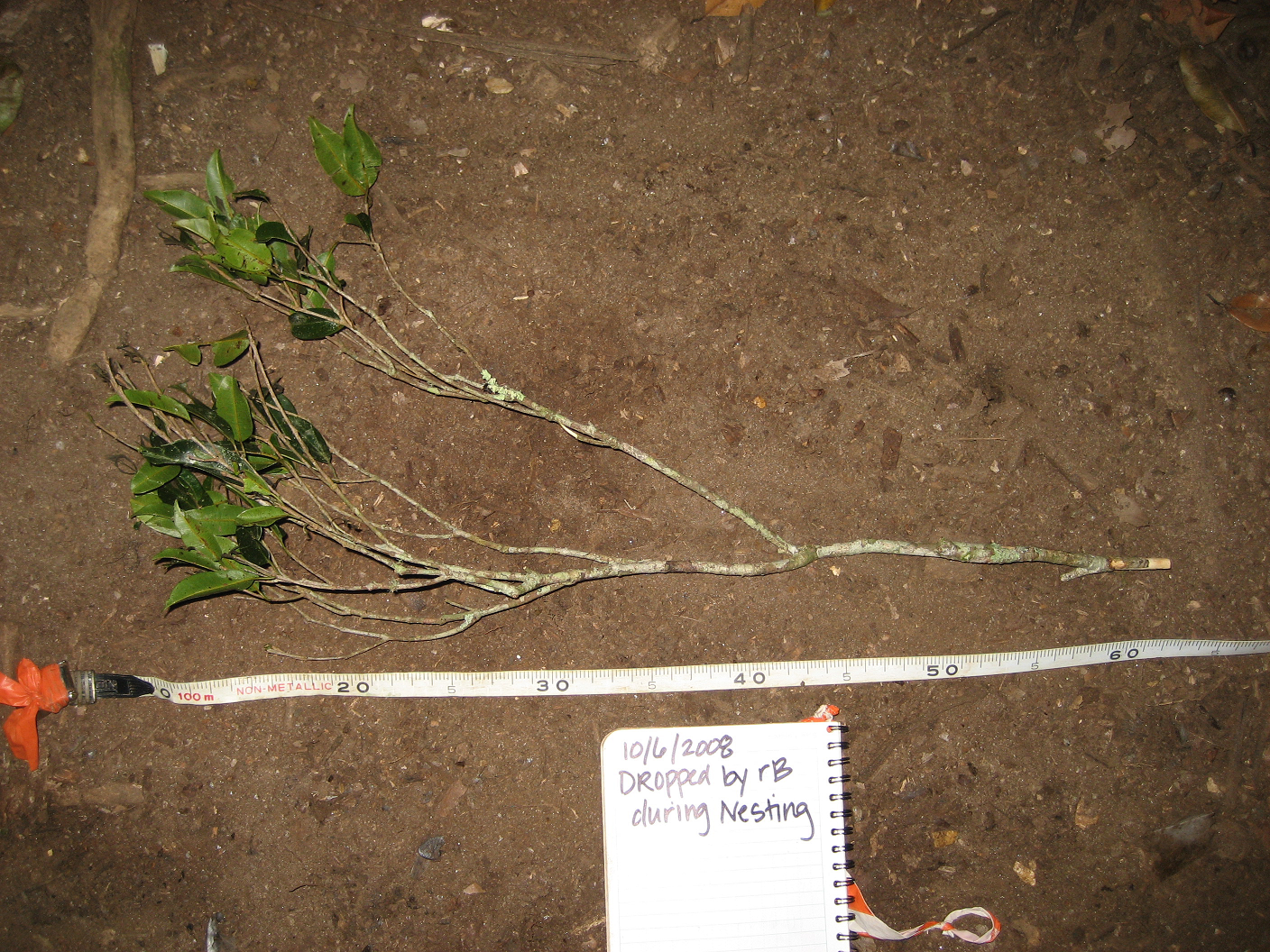

Supplement: Supplementary file 1 [file ECE3-9-1010-s001.tif]
